# Current Understanding of Polyphenols to Enhance Bioavailability for Better Therapies

**DOI:** 10.3390/biomedicines11072078

**Published:** 2023-07-24

**Authors:** Mohammad Aatif

**Affiliations:** Department of Public Health, College of Applied Medical Sciences, King Faisal University, Al Ahsa 31982, Saudi Arabia; maahmad@kfu.edu.sa

**Keywords:** polyphenols, bioavailability, nanocarriers, molecular mechanisms, human health

## Abstract

In recent years, plant polyphenols have become a popular focus for the development of novel functional foods. Polyphenols, a class of bioactive compounds, including flavonoids, phenolic acids, and lignans, are commonly found in plant-based diets with a variety of biological actions, including antioxidant, anti-inflammatory, and anticancer effects. Unfortunately, polyphenols are not widely used in nutraceuticals since many of the chemicals in polyphenols possess poor oral bioavailability. Thankfully, polyphenols can be encapsulated and transported using bio-based nanocarriers, thereby increasing their bioavailability. Polyphenols’ limited water solubility and low bioavailability are limiting factors for their practical usage, but this issue can be resolved if suitable delivery vehicles are developed for encapsulating and delivering polyphenolic compounds. This paper provides an overview of the study of nanocarriers for the enhancement of polyphenol oral bioavailability, as well as a summary of the health advantages of polyphenols in the prevention and treatment of several diseases.

## 1. Introduction

The health benefits of plant-based functional foods have attracted a growing amount of scientific interest, and plant polyphenols, one of the most common chemical components of plants, have particular attention. A diet full of vegetables, fruits, grains, tea, and coffee contains polyphenols in their natural forms. Polyphenolic compounds have a wide range of varied structures based on phenolic rings [1,2] and can be broken down into phenolic acids, flavonoids, anthocyanins, and tannins. Several foods consist of bioactive chemicals or molecules that have a biological effect on the body. Polyphenols are one type of bioactive compound found in plant diets [2,3]. Some polyphenols can be obtained through reprocessing natural substances, while others can be separated and extracted straight from natural foods. The chemical composition of polyphenols determines their rate of absorption and the extent of their action in the digestive tract [4]. Because of their unique chemical composition, polyphenols can serve as effective preventative agents against chronic and degenerative diseases [3,4,5]. The protective effects of polyphenols against cardiovascular disease, neurological disease, liver disease, diabetes, and cancer have been demonstrated by a number of researchers [6,7,8,9,10,11,12].

Polyphenols have been shown in multiple research to be helpful to intestinal health and to have beneficial properties, such as lowering inflammation and protecting against cancer through controlling the gut flora [2,13]. This is why they are frequently included in functional diet menus, which aim to provide health benefits through ingestion. As the global population grows and the awareness of the need to maintain a healthy lifestyle expands, so does the demand for polyphenols in the market. The use of polyphenols in nutraceuticals is hindered, however, because many polyphenolic substances have poor oral bioavailability.

**Figure 1:**
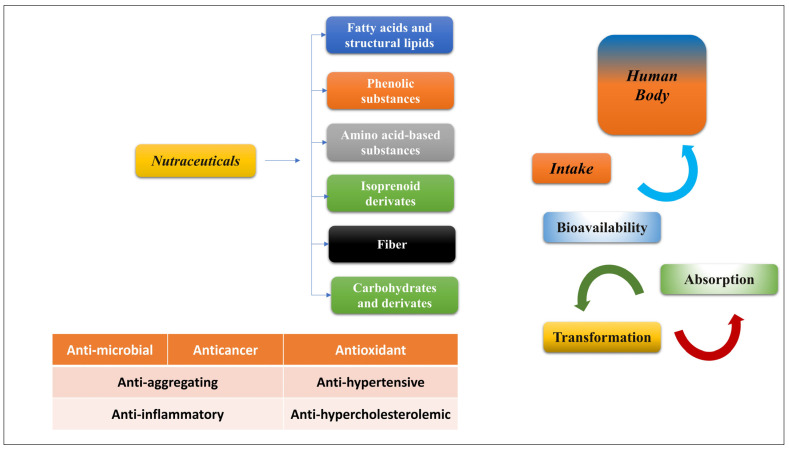
Beneficial health effects of polyphenols on human health.

Natural bio-based nanocarriers are the ideal choice for enclosing, preserving, and transporting polyphenols, which increases their bioavailability (Figure 1) [2,14]. To overcome the problems associated with the poor oral absorption of polyphenols, bio-based polymers (such as those containing proteins and polysaccharides) are appropriate delivery systems when employed as nutritional treatments since they are biocompatible, biodegradable, resource-sustainable, and nutritionally valuable [2,14]. Protein bio-based polymers possess a wide range of useful features, such as those of emulsification, amphiphilicity, gelation, and foaming, in addition to their high nutritional value [14]. Because of their typical or distinctive molecular structure and functional properties, they may be fabricated into a wide variety of nanoscale delivery vehicles, including nanoparticles, nanogels, nano-emulsions, nanofilms, and nanofibers, that can transport both hydrophilic and hydrophobic polyphenolic chemicals [14,15]. Bio-based polymeric polysaccharides can be used as building blocks in the production of various nanocarriers and in the transport of polyphenolic compounds [1,2]. As they are both biocompatible and biodegradable, lipid-based nanocarriers have emerged as a key technology for delivering polyphenols in the food and nutrition industry [2]. To further investigate and create innovative polyphenol health nutrition products [2,15], some polyphenol products have been put into an effort to further develop novel polyphenol-based health nutrition products [2,15]. Lipid-based delivery vectors, such as liposomes, nano-emulsions, and solid lipid nanoparticles, protect fat-soluble polyphenols from degradation in the digestive tract and increase their bioavailability [1,2,4,12,15].

This article provides a general introduction to the classification of polyphenolic chemicals and a comprehensive evaluation of the positive effects of polyphenols on human health. The bioavailability of polyphenolic compounds is low, but in this article, possible solutions to this problem are suggested, including the development and use of food-grade bio-based nanocarriers to encapsulate, preserve, and distribute these compounds. Finally, the potential use of polyphenol food-grade bio-based nano-complexes in nutraceuticals is exciting, as it promises to shed light on the creation and use of nutraceuticals by bringing together nano-delivery techniques with polyphenol health advantages.

## 2. Polyphenols in Plants: A Review of Their Taxonomy, Properties, and Health Benefits

### 2.1. Categorization and Characteristics

Many different foods include plant polyphenols, which have received a lot of attention due to their potential for various biological effects. Chemicals with a benzene ring structure and two or more phenolic hydroxyl groups are known as polyphenols [1,2,15]. These compounds can be further categorized into flavonoids and phenolic acids based on their structures.

**Figure 2:**
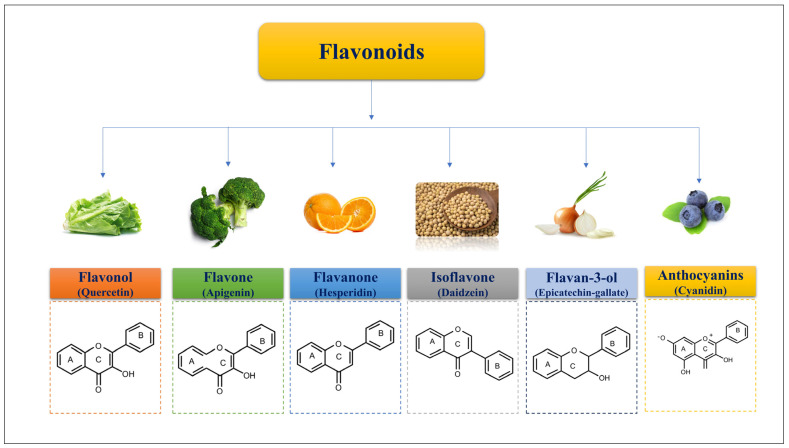
Flavonoid subclasses and their representative structures with dietary examples.

Flavonoids are primarily found in plant cells as glycosides housed in vesicles. Flavonoids consist of a C6-C3-C6 tricyclic structure in their molecular core. Flavonoids are even further categorized into subcategories based on their chemical structures [2], such as flavonols, flavones, isoflavones, anthocyanins, flavanones, and flavanols (Figure 2). Flavonoids, found in most plants, have been shown to play a crucial role in plant development, growth, flowering, fruiting, antibacterial action, and disease prevention [2]. Human health may benefit from the majority of these flavonoids due to their anti-inflammatory, antibacterial, antioxidant, and anticancer physiological actions [12].

Fruits, vegetables, and certain drinks contain phenolic acids. It is reported that low-molecular-weight phenolic acids are water-soluble; however, after enduring condensation reactions with glucose and quinic acid, they become water-insoluble, resulting in poor bioavailability [13,16]. The above-mentioned biological characteristics of phenolic acids have led to their widespread usage in the production of functional foods [17].

### 2.2. Beneficial Effects

Polyphenols found in plants have several positive benefits on human health. Because of their high levels of antioxidants and antibacterial qualities, as well as their availability and biocompatibility, they can be added to foods and endowed with special functional features that have a positive impact on people’s health. Table 1 shows the possible application of polyphenols in food formulations by presenting some of the beneficial effects of plant polyphenols on human health. Important steps toward reducing the prevalence of diabetes, high blood pressure, and cancer could be taken with the help of polyphenolic-compound-containing functional foods.

### 2.3. Antioxidant Effects

The unique structural properties of plant polyphenolic compounds are responsible for their potent antioxidant activity. Antioxidants like polyphenols can protect DNA from oxidative damage, a leading cause of many illnesses. In vitro studies have revealed that EGCG has a cancer-preventive effect by decreasing the formation of ROS in the body and that EGCG can speed up programmed cell death by limiting DNA synthesis in cancer cells while not affecting normal cells [2,3]. EGCG reduces the growth of human colon cancer cells HCT-116 and SW-480, but EGCG has the strongest effect, which is mostly due to its phenolic content [2,3]. EGCG regulates oxidative-stress-induced apoptosis via the protein kinase B (Akt) and c-Jun N-terminal kinase (JNK) signaling pathways in the cell signaling system [2]. EGCG increases the expression of mitogen-activated protein kinase (MAPK) and antioxidant response element (ARE) genes, boosting the ability of the cell’s antioxidant defense system [2,3]. Furthermore, nuclear factor erythroid 2-related factor 2 (Nrf2), nuclear factor-kappa B (NF-κB), and others are important cellular pathways for the body’s antioxidants [2,3]. In addition, EGCG has been proven in vivo to raise serum catalase (CAT), glutathione peroxidase (GSH-Px), and superoxide dismutase (SOD) levels while decreasing MDA generation [2,21]. From the available databases, Farhan et al. [2] looked for randomized clinical trials and reported that patients undergoing radiation therapy for esophageal cancer who also suffer from acute radiation-induced esophagitis (ARIE) may benefit from taking an EGCG solution orally, according to clinical findings from a study [36]. Although EGCG does not improve radiation efficacy, it may act as a preventative measure against other potential side effects. [36]. A once-daily oral dosage of EGCG in the Polyphenon E formulation was reported to be safe and well tolerated in a phase 2 trial for patients with chronic lymphocytic leukemia (CLL) [37]. Long-term improvements in absolute lymphocyte count (ALC) and/or lymphadenopathy were seen in the vast majority of individuals. Oral EGCG formulations that are more likely to be absorbed by the body are now under development and may be more effective [37]. A standardized catechin mixture containing EGCG at 200 mg BID for 12 months was well tolerated and accumulated in plasma [38], but it did not reduce the risk of a subsequent diagnosis of prostate cancer in men with baseline high-grade prostatic intraepithelial neoplasia (HGPIN) and atypical small acinar proliferation (ASAP). According to the study [38], a biopsy of the prostate taken within a year after an HGPIN diagnosis has only a 20% chance of revealing cancer if an adequate sample is performed at the outset. In addition, the low rate of prostate cancer at one year observed in men with ASAP in this trial suggests that earlier findings may have exaggerated the underlying risk of cancer in that cohort. Based on the studies and available literature, it is safe to assume that plant polyphenols possess potent antioxidant properties that may be exploited for human benefit [2].

### 2.4. Anti-Inflammatory Effects

Plant polyphenols inhibit and kill some inflammatory cells via interacting with cytokines and their receptors or by changing the release of cytokines. According to the research [39], rutin-containing hydrogels exhibited anti-inflammatory effectiveness that was on par with that of conventional medicines. Researchers have examined hesperidin’s anti-inflammatory properties, which were evaluated using RAW264.7 cells and a CCl4-induced acute liver injury model [40]; the results showed that the compound significantly inhibited the production of nitric oxide (NO), interleukin 6 (IL-6), and tumor necrosis factor-alpha (TNF-α), both in vivo and in vitro. Studies show and confirm that polyphenols certainly have the properties to protect the body from inflammation [2].

### 2.5. Anti-Cancer Effect

Certain forms of cancer are more vulnerable to polyphenols’ preventive effects. In addition to preventing tumor growth, their toxic effects on cells can trigger apoptosis. A study [41] found that resveratrol has an inhibitory effect on cell growth, an apoptotic effect, and good antioxidant characteristics, all of which can alter the course of cancer and related disorders. In addition, quercetin’s widespread use in the treatment and prevention of esophageal cancer was highlighted in the report [41]. Several other investigations have found that polyphenolic substances, such as EGCG, genistein, naringin, and curcumin, have anticancer effects [18,20,24,42]. These polyphenols can eliminate cancer cells by altering signaling pathways, blocking cell cycle events, and inducing apoptosis, among other anticancer strategies. Tumor cell proliferation enzymes are another target of polyphenol regulation. Recent research is pointing to natural polyphenols and their anti-cancer potential via multiple mechanisms, including their ability to inhibit angiogenesis, metastasis, and DNA interaction [2,3,31,33,37,38].

### 2.6. Anti-Microbial Effect

Polyphenols are effective antimicrobial agents against many different types of bacteria. The antibacterial activity of flavonoids is exceptionally high in comparison to that of other polyphenolic substances. Polyphenolic substances, according to some studies, can work in tandem with antibiotics to boost their efficacy against bacteria. Curcumin placed onto chitosan films exhibited strong antibacterial activity against *Staphylococcus aureus* and *Rhizopus solani* [34,43]. Tea polyphenols (EGCG, EGC, ECG), silymarin, and rutin have all shown antibacterial activity, and their use has been widely reported and confirmed in various studies [44,45]. The above polyphenols lowered cell viability, extracellular DNA, and exopolysaccharide levels. Bacterial adherence to human keratinocytes was shown to be moderately reduced after treatment. Membrane permeability was another mechanism impeded by these polyphenols [43,44,45,46].

### 2.7. Pro-Oxidant Effect

The antioxidant effects of polyphenolic substances are particularly noteworthy. High dosages of certain polyphenolic chemicals, however, have been shown to cause DNA damage and ultimately apoptosis [47]. Hadi et al. [48] have studied the pro-oxidant phenomenon of polyphenols for the past three and a half decades. The majority of polyphenols found in plants can function as antioxidants or pro-oxidants. Several studies have shown that polyphenolic compounds, when combined with copper ions, can function as pro-oxidants, causing DNA damage in cells via the production of reactive oxygen species (ROS) [49,50,51,52,53]. Several polyphenols (such as EGCG, quercetin, resveratrol, and daidzein) have been reported to elicit this effect via intracellular copper ion mobilization [50,51,52,53,54,55,56]. The pro-oxidant behavior, therefore, could be a useful weapon to selectively target cancer cells while sparing normal cells for designing safer cancer therapeutics.

### 2.8. Antidiabetic Effect

Polyphenol-rich diets may help lower diabetes risk. Peripheral tissue insulin sensitivity may be improved by polyphenols, according to several studies [57]. Alpha-amylase and alpha-glucosidase, which control intestinal glucose absorption and blood sugar regulation, are strongly inhibited by many polyphenolic substances [58]. Catechins, a type of polyphenolic chemical found in foods like tea, are known to have powerful antioxidant and anti-diabetic effects. In a recent review [59], the therapeutic potential of quercetin as an antidiabetic bioactive component was reported in depth. It was explained that quercetin has good preventive and therapeutic potential against diabetes in vitro and in vivo and that this potential is supported by a complete and systematic summary and description of the mechanism of action, the targets, and the effects of quercetin [59]. As a result, polyphenolic chemicals show considerable promise as a tool for halting the development of diabetes.

### 2.9. Anti-Hypertensive Effect

Improved endothelial function, decreased oxidative sensitivity of low-density lipoproteins, and increased vasodilation have all been linked to polyphenolic chemicals, such as those found in cocoa, which is high in flavanol compounds, like catechins and proanthocyanidins. The European Food Safety Authority (EFSA) [60] acknowledges this effect of polyphenols thoroughly. Various polyphenolic substances have been shown to have positive effects on vasodilation and other aspects of blood pressure regulation, as discussed in detail [61]. In a study, it was found that curcumin, amlodipine, and a combination of the two all had vasodilatory effects on isolated rat aortic rings. The combined administration of curcumin and amlodipine induced a stronger vasorelaxant effect than amlodipine alone [62]. This suggests that people with high blood pressure who take amlodipine could safely consume curcumin for food or other medical reasons without affecting the antihypertensive effects of the drug [62].

## 3. Polyphenol Bioavailability

There is no correlation between the quantity of polyphenols in food and their bioavailability. When taken orally, polyphenols gain access to the bloodstream via the intestinal mucosa and then travel to the intended tissues. A number of studies confirm the rising interest in nutraceuticals [63]. The regulation of several metabolic functions is profoundly influenced by one’s dietary habits. Foods are more than just fuel for the body’s metabolic processes; they also contain “active substances” that have positive impacts on health, such as antioxidants, vitamins, polyunsaturated fatty acids, and fiber. Hence, a healthy diet and all of its constituents can help people feel better, lower their chances of developing certain diseases, and generally increase their quality of life [64,65,66].

The most studied classes of physiologically active molecules include fatty acids and lipids, amino-acid-based substances, carbohydrates, fiber, isoprenoid derivatives, and phenolic compounds (Figure 1). The intestinal absorption and bio-accessibility of these nutritious substances [67,68,69] are crucial factors to think about when assessing their bioavailability. For instance, polyphenols have a modulating influence on the gut microbiota by blocking pathogenic bacteria and increasing good bacteria, both of which have positive effects on host health. The mucus layer, made up of epithelial cells, can act as a barrier for some nutraceutical chemicals, limiting their absorption after ingestion [70]. Nevertheless, bioactive chemicals exert favorable impacts on human health when they are taken up from food and become soluble in gastrointestinal fluids.

Microencapsulated bioactive molecules, like flavonoids, phenolic compounds, antioxidant molecules, carotenoids, and general plant metabolites, are examples of bioavailable nutraceutical chemicals. In reality, active substances, including pigments, antioxidants, vitamins, minerals, peptides, and proteins, are protected, stabilized, and their bioavailability is increased and controlled through the use of micro/nano-encapsulation procedures. By encapsulating them, nutraceuticals are better able to withstand the rigors of the digestive system, interact with it, and remain soluble and bioavailable [71,72,73].

Certain fruits, such as apples and berries, contain more than 200 mg of polyphenols per 100 g of fresh fruit [74]. The amount and bioavailability of these phenolic chemicals, however, are affected by the method(s) of food processing used. For instance, since many food-processing techniques require heat treatment, it is commonly seen that exposure to higher temperatures might negatively affect the nutritional profile of fruits and vegetables; nevertheless, some research has found the opposite to be true [75]. Domestic cooking was found to cause significant losses in polyphenols, with wide variation among meals, according to a comprehensive investigation of ~161 polyphenols and their food-processing alterations. Furthermore, it was found that the food under study was frequently more influential than the procedure used, demonstrating the significance of the food matrix [76]. The fate of polyphenolic compounds during food processing depends on a wide variety of parameters, including the type of food-processing technique employed in both commercial and home kitchens, and these correlations are briefly discussed here.

Both at home and in large-scale food-processing facilities, heat treatments are in practice. Cooking methods, such as the stovetop, the microwave, and the steam oven, can be used for these preparations. Toasting, coffee roasting, drying, canning, pasteurizing, and sterilizing are all examples of additional common transformation methods that rely on heat. What happens to polyphenols upon heating depends on the processing technique used. By destroying cell walls, heat increases the availability of phenolic compounds that are bound elsewhere in the plant [77]. They are more susceptible to oxidation, though, and only a few of them are truly thermostable. It is observed that the polyphenol profiles of foods vary depending on how long they are boiled. For instance, kale leaves lost 51% of their polyphenol content after being blanched (short-interval boiled), with the loss of caffeic acid being the lowest (28%), and the loss of ferulic acid being the greatest (55%). However, the total polyphenol content decreased by 73% because of the more severe damage caused by extended cooking times [78].

Canning is a method for economically producing microbiologically safe and sterile products through the use of heat treatment. Retorts, pasteurizers, and heat exchangers are all suitable for processing food into the canned form. The primary goal of heat treatment is to kill off any potentially harmful bacteria, and metal cans, glass jars, and retort pouches are employed to keep out any potential spoilers. Both the food and the jar can be kept in good condition if they are cooled to room temperature after being heated [79]. When phenolic components leach (draining process) into the surrounding medium (brine or syrup), total phenolic and flavonoid concentrations are said to decrease during canning [80]. The applied heat treatment disintegrates the cells and tissues, allowing the polyphenols to migrate into the media and cause this widespread leaching.

The development of water crystals and ice at temperatures below freezing is a strategy used for preservation because it slows down biological and physicochemical events. Hence, it inhibits the growth of pathogenic microbes and extends the storage life of food [81]. To a lesser extent (in a temperature range of 1 to 8 °C), chilling or cooling meals decreases microbiological and metabolic changes to maintain stability. Foods that have been cooled from their original cooking temperature keep their quality for longer [81]. According to a study, blanching or cooking kale leaves prior to freezing did not have a significant effect on their bioavailability [78].

To enhance the bioavailability and health advantages of dietary polyphenols, it will be crucial for future studies and reviews to also examine the possibility of other techniques on beneficial impacts on processing conditions according to each food matrix [82].

## 4. Nanoformulations Made from Dietary Macromolecules to Encapsulate and Transport Polyphenols

**Figure 3:**
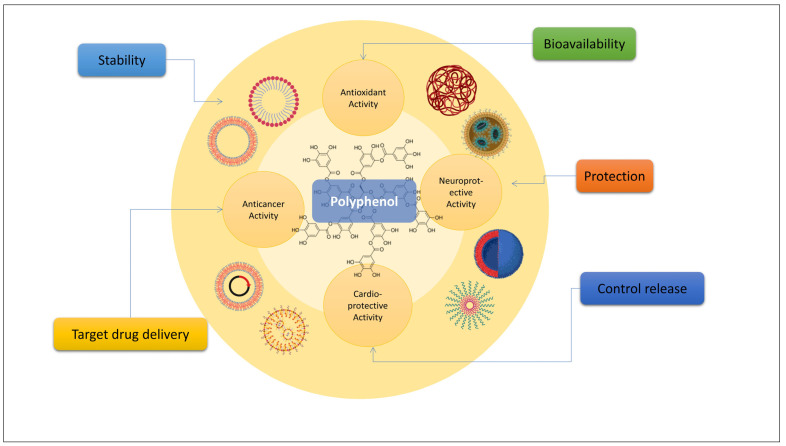
Advantages of using polyphenol-based nanoformulations.

Dietary proteins have the unique property of having an exceptional binding ability with various pharmaceuticals or nutraceuticals, making them an ideal renewable raw material for constructing nanocarriers for medication or nutraceutical delivery (Figure 3). Proteins found in food have many health benefits and advantages, such as being antigenic and biodegradable. Protein nanoparticles have a low barrier to entry in terms of synthesis and production scale [83,84]. Some of the most often used food-grade proteins in the development of nanoparticle delivery systems for the encapsulation of dietary polyphenols are reviewed and discussed below. These might open doors for the better absorption of polyphenols in the human body.

### 4.1. Nanoformulations of Casein

Proline-rich, open-structured rheomorphic caseins exhibit separate hydrophobic and hydrophilic domains. Caseins (95%) are spontaneously self-assembled into casein micelles, which are spherical colloidal particles with sizes of 50–500 nm (average 150 nm). Hydrophobic interactions improved curcumin’s solubility at least 2500-fold in camel β-casein micelles. Curcumin in a β-casein micelle has more antioxidant activity than free β-casein and curcumin. The human leukemia cell line K-562 was more sensitive to encapsulated curcumin than free curcumin [85]. A group studied the spray-dried curcumin-loaded casein nanoparticles from a warm aqueous ethanol solution with dissolved sodium caseinate and curcumin [86]. Curcumin with casein nanoparticles was a more potent antioxidant and cytotoxic than virgin curcumin. The researchers have proposed a low-cost, low-energy, organic solvent-free encapsulation method based on curcumin’s pH-dependent solubility and sodium caseinate’s self-assembly [86]. Curcumin was encapsulated in self-assembled casein nanoparticles after neutralization at pH 12 and 21 °C. Casein nanoparticle-encapsulated curcumin boosted human colorectal and pancreatic cancer cell growth [86]. Another research group [87] observed that attaching casein to curcumin at pH 7.2 increased its stability. Curcumin’s hemolysis prevention activity was unaffected by the curcumin–casein interaction [87]. This shows a very promising method of polyphenol absorption.

### 4.2. Nanoformulations of Gelatin

Gelatin is a collagen that is denatured via acid and alkaline hydrolysis. It has been used safely in pharmaceuticals, cosmetics, and food goods for years by the Food and Drug Administration. It is observed that EGCG encapsulated in gelatin-based nanoparticles blocked hepatocyte growth factor-induced intracellular signaling in MBA-MD-231 breast cancer cells as well as free EGCG [88]. It was also observed that coculture of high-loading resveratrol-loaded gelatin nanoparticles caused cell death by altering p53, p21, caspase-3, Bax, Bcl-2, and NF-κB expression [89]. Based on the observations, it is indeed possible to use polyphenols in combination with gelatin for treating deadly diseases such as cancer.

### 4.3. Nanoformulations of Polysaccharides in Food

Polysaccharides contribute to food texture, flavor, and caloric value. Glycosidic linkages link monosaccharides to polysaccharides. Polysaccharides’ bio adhesion, especially for mucosal surfaces, has been employed to target organs or cells and lengthen polyphenol residency time in the colon. Chitosan is the widely used polysaccharide in oral administration nanoparticle systems [90]. The most extensively disseminated biopolymer is a cationic, nontoxic, biodegradable, and biocompatible polyelectrolyte with an oral LD50 in mice of over 16 g/kg [90]. Japan, Italy, and Finland have approved it for dietary use. It improves the intestinal absorption of active substances, especially water-soluble molecules like EGCG with low small intestine permeability. Chitosan interacts well with negatively charged polymers and can be modified with various functional groups to give nanoparticle-targeting properties [90].

### 4.4. Nanoformulations with a Protein–Polysaccharide Conjugate

The Maillard process produces polysaccharide glycosylated proteins that limit protein precipitation induced by the high concentration or contact with polyphenols, which are polyphenol encapsulation materials [91]. The protein core of Maillard-synthesized gelatin–dextran conjugate nanoparticles included tea polyphenols. EGCG-loaded conjugate nanoparticles had an average diameter of 86 nm and limited distribution under optimum circumstances. EGCG has a 360 wt.% loading capacity and pH-independent encapsulation efficiency. Encapsulated EGCG had equivalent or higher cytotoxicity against MCF-7 cells than free EGCG in 3-(4,5-dimethylthiazol-2-yl)-2,5-diphenyltetrazolium bromide (MTT) assay [92]. Dextran-glycosylated casein nanoparticles contained and retained EGCG and had great colloid stability in a wide concentration range during storage. Glycosylated casein protected EGCG in alkaline pH and released slowly in intestinal fluid [92]. The Maillard reaction conjugated dextran to bovine serum albumin to give EGCG for protein glycosylation [93].

### 4.5. Nanoformulations Derived from Dietary Lipids

Solid lipid nanoparticles (SLNs) improve lipid-soluble polyphenol solubility and bioavailability. In a coculture system of absorptive Caco-2 and mucus-secreting HT29-MTX cells, SLN’s curcumin delivery was tested, and it was found that curcumin encapsulated in SLN delivered better than unencapsulated curcumin without affecting cellular junction integrity [94]. Another study [95] found that curcumin-loaded SLNs could prolong in vitro anticancer efficacy, cellular absorption, and in vivo bioavailability. Resveratrol was loaded and encapsulated in two forms of SLN [96]. The nanoparticle crystal structure was changed by resveratrol, suggesting its entrapment. During incubation in digestive fluids, resveratrol mainly stayed with lipid nanoparticles [96]. A study [97] created glyceryl behenate-based solid SLNs to encapsulate and distribute resveratrol. Resveratrol-loaded SLNs were as effective as free resveratrol as an anticancer drug in a cytotoxicity experiment. In a Wistar rat bio-distribution investigation, SLNs increased brain resveratrol content by *p* < 0.001 [97]. Resveratrol-loaded stearic-acid-based SLNs coated with poloxamer 188 were successfully synthesized utilizing solvent diffusion–solvent evaporation and showed extended drug release in vitro up to 120 h. Compared to the solution, the lipid formulation improved the oral bioavailability of resveratrol by eight-fold [98]. After loading into SLNs, resveratrol solubility, stability, and intracellular delivery increased. With or without resveratrol, SLNs below 180 nm loading went quickly across the cell membrane, diffused throughout the cytoplasm, migrated sequentially among cellular levels, and localized in the perinuclear region without cytotoxicity. Resveratrol in solution was less cytostatic than SLN–resveratrol. Resveratrol’s cell-proliferation-reducing effects may be enhanced by SLN delivery [99]. Therefore, it can be concluded that nanoformulations based on dietary lipids can be treated as a reliable vehicle delivery for polyphenols.

Therefore, it may be concluded that polysaccharides, whether isolated or attached to proteins, are commonly employed for the nanoencapsulation of quercetin, EGCG, and resveratrol, as summarized in Table 2. These polyphenolic compounds were nanoencapsulated to maintain their physicochemical stability and characteristics, such as antibacterial, anticancer, antioxidant, and antiproliferative activity. Furthermore, the nanoencapsulation process can improve the intestinal release rate, bioaccessibility, and bioavailability, inhibit gastrointestinal degradation, ensure storage stability and color protection, apply in food packaging, promote thermal stability, and allow for oral administration of these compounds.

## 5. Nanoformulations of Polyphenols and Therapeutic Properties

### 5.1. Cardioprotective Effects

Oxidative stress is thought to be a major factor in the development of cardiovascular disease. Increased oxidative stress and a diminished antioxidant reserve are associated with both acute and chronic heart failure [100]. Cardiovascular disease, especially ischemic heart disease, and stroke due to atherosclerosis are among the oxidative-related disorders that polyphenols can protect. Polyphenols have been shown to protect the heart against oxidative stress-related diseases in a number of studies [101,102]. A bioactive polymer (PLGA layer) was deposited on top of a superparamagnetic SiN.SiN@QC-PLGA nano-bio-composite [103] to modify the drug discharge profile and increase the functional resemblance to the local myocardial by facilitating the cell recruitment, expansion, attachment, and articulation of cardiac proteins. The effectiveness of recently produced nano-formulated natural therapies against hypertension, atherosclerosis, thrombosis, and myocardial infarction has been studied [104]. The protective effects of a curcumin nanoformulation in cardiomyocytes were studied recently [105]. Cur-Res-mP127 (co-loaded curcumin and resveratrol at a molar ratio of 5:1 in Pluronic^®^ F127 micelles) was found to be cardioprotective in a rat embryonic cardiomyocyte (H9C2) model by reducing apoptosis and reactive oxygen species (ROS) [105]. As shown in Table 3, polyphenols loaded with nanoparticles using conjugation and encapsulation techniques are effective in treating a variety of disorders and diseases.

### 5.2. Neuroprotective Effects

In oxidative stress, the high quantities of free radicals produced overwhelm the antioxidant system. Superoxide anions, hydroxyl radicals, hydrogen peroxide radicals, and peroxyl radicals are all examples of reactive oxygen species (O_2_). Since it is the most oxidative organ in the body, the brain uses 20% of the total basal oxygen and is subjected to one of the highest levels of oxidative stress [106]. Endogenous processes that detoxify oxidative damage include catalase, glutathione/glutathione peroxidase, superoxide dismutase, and vitamins E and C, among others. In many diseases, however, oxidative stress is exacerbated because free radical production exceeds the body’s defenses. Most neurodegenerative diseases are associated with high levels of oxidative stress in the brain [107]. These diseases include Parkinson’s, Alzheimer’s, Huntington’s, multiple sclerosis, traumatic brain injury, ischemia, and aging. Polyphenols have been shown to have neuroprotective benefits by lowering oxidative stress in the brain [108]. Cerium oxide nanoparticles are being evaluated for use in biomedicine due to their strong regenerative antioxidant qualities, which have led to their widespread use in the materials sector. Researchers are studying cerium oxide nanoparticles because they show promising molecules as a treatment for several neurological illnesses [109]. The neuroprotective effects of CeO_2_@SiO_2_-PEG nanoparticles (CSP-NPs) for proanthocyanidin and curcumin delivery have been studied [110]. Hydrophilic curcumin (Cur) and hydrophobic proanthocyanidin (PAC) were, respectively, loaded onto CeO_2_@SiO_2_-PEG nanoparticles to produce Cur-NPs and PAC-NPs. Cur-NPs and PAC-NPs inhibited acetylcholinesterase (AchE) activity and protected neurons against A1-42-mediated toxicity in PC-12 cells. Several studies have reported different nanoparticle systems loaded with curcumin. These systems include poly (-caprolactone) (PCL), poly (lactide-co-glycolide) (PLGA), and methoxy poly (ethylene glycol) poly (-caprolactone) (MPEG-PCL). Inhibiting enzymatic and pH degradation of curcumin and showcasing its neuroprotective capabilities [111] are both made possible through the incorporation of curcumin into nanoparticle systems. A safe and efficient therapeutic approach for the treatment of Alzheimer’s disease may be the development of PEGylated PLGA nanoparticles loaded with two medicines (EGCG and acetyl acid). When mice were orally administered EGCG/ascorbic acid NPs, the compound accumulated throughout the brain and other important organs. This formulation has been shown to have the potential to increase the drug’s persistence in both the blood and the brain [112]. Research has found that 4-hydroxyisophthalic acid (4-HIA)-encapsulated PLGA-NPs significantly reduced the cytotoxicity of H_2_O_2_ in PC12 cells [113]. Thus, the neuroprotective effects are well established using nanoformulations and polyphenols.

### 5.3. Cancer Treatment

Cancer is defined as an uncontrolled growth of cells, which can lead to malignancies that are both potentially fatal and extremely costly for patients and the healthcare system. Many different diseases have traditionally been treated and prevented with natural polyphenols. Hence, due to their anticancer effects, these phytochemicals may be used as chemotherapeutic and chemopreventive drugs in a variety of malignancies [114]. The cytotoxic effects of curcumin-loaded PLGA nanoparticles coupled with anti-P-glycoprotein were studied in human cervical cancer KB-3-1 and KB-V1 cells, and the results showed increased curcumin solubility and cellular absorption, as well as decreased cell survival [115]. To activate PTT-assisted ferrous therapy in the treatment of cancer, the authors of [116] designed ferric-coordinated polyphenol nanoparticles. Ellagic acid loaded with schizophyllan and chitin nanoparticles exhibits anticancer effects [117] in MCF-7 breast cancer cells. Viability assays showed that MCF-7 cells were significantly inhibited in their ability to proliferate, with the impact being amplified at higher concentrations. The nanoencapsulation of quercetin and curcumin in a casein-based model was recently described [118], and these compounds were evaluated against MCF-7 cell lines. Here, tumor cell growth was slowed by the encapsulated polyphenols, more so than the unencapsulated ones. To be specific, the cerium nanoparticles made using the green approach contained all the hallmarks of a functioning nanoparticle and increased the expression of the genes for the two main antioxidant-related enzymes, catalase (CAT) and superoxide dismutase (SOD). Cerium oxide nanoparticles (CeO-NPs) showed increased cytotoxicity against breast cancer cells compared to normal cells. As CeO-NPs protected healthy cells from oxidative stress and inflammation caused by free radicals, appearing to be a promising therapeutic agent for the treatment of breast cancer cells [119].

**Table 3:** Polyphenol-loaded nanoformulations and disorders cured.

Polyphenol	Type of Disease	Nanoformulation	References
Curcumin	Cancer	PLGA	[115]
Ellagic acid	Cancer	Schizophyllan and chitin nanoparticles	[117]
Quercetin	Cancer	Nanocapsules	[118]
Resveratrol	Cardiovascular	Pluronic^®^ F127	[105]
4-hydroxyisophthalic acid	Neurological	Polymeric nanocarrier (PLGA)-NPs	[109]
Proanthocyanidins and curcumin	Neurological	CeO_2_@SiO_2_-PEG nanoparticles (CSP-NPs)	[110]
Naringenin	Neurological	PLGA: PCL-gelatin-coated	[120]
Gallic acid	Neurological	Lipid nanocarrier: GA-NPs	[121]
EGCG	Skin Inflammation	Polymeric nanocarrier: PEG-PLGA	[122]
Caffeic acid	Skin Inflammation	Nanofiber nanocarrier: PLGA	[123]
Apigenin	Skin Inflammation	Ethosomal nanocarrier	[124]
Curcumin	Cardiovascular	Chitosan NPs	[125]
Quercetin	Cardiovascular	PLGA NPs	[126]

## 6. Polyphenol-Based Nanoformulations: Takeaway Message

Several important concerns need to be addressed in the future to speed up the development and clinical translation of polyphenol-containing nanoformulations: (a) The manufacture of polyphenol-containing nanoformulations requires the development of simple and generic methodologies, as well as the actualization of rational design and on-demand synthesis. It is important to learn more about the use of the materials’ qualities and functionalities. In their current form, nanoformulations containing polyphenols have a number of drawbacks, including a lack of physiological stability/biodegradability, drug encapsulation/loading efficiency, stimulus responsiveness, traceability, and active targeting ability. Some fresh perspectives could emerge from combining chemical grafting with supramolecular self-assembly. (b) Cancer combination therapy has found an excellent new platform in polyphenol-containing nanoformulations. Better anticancer tactics may be attainable through the development of multifunctional polyphenol-based nanoplatforms that integrate various cancer therapeutic modalities (such as chemotherapy, radiation, and immunotherapy). Nanoformulations containing polyphenols have many potential medical uses, but further research is needed. While most research so far has been on cancer treatment, polyphenols’ many health benefits also make them useful in preventing and managing bacterial infection, neurological diseases, cardiovascular conditions, diabetes, and others. Furthermore, it is anticipated that the polyphenol-containing nanoformulations will be able to include different enzymes for biocatalysis purposes. Imaging agents (fluorescent probes, MRI agents, radioactive agents) should be incorporated into various illness therapies with increased focus. (c) Systematic assessments of the biosafety and in vivo destiny of polyphenol-containing nanoformulations are also crucial. Research into polyphenol-containing nanoformulations should focus on their targeting abilities to tumor or inflamed tissues, long-term toxicity, in vivo biodegradability, renal clearance, and interaction processes with biological systems.

To ensure their use in safe therapeutic settings, understanding how these polyphenol-containing materials interact with blood, immunological, and normal tissue cells is essential. Additionally, in the future, more biosafety analyses should be performed utilizing animal models. In vitro or pilot animal trials have, thus far, dominated research on polyphenol-containing nanoformulations. Carcinogenicity, genotoxicity, mutagenicity, reproductive toxicity, and teratogenicity of polyphenol-containing nanoformulations, among other clinically important indices, are mostly unknown.

Currently, the most used methods, like ionic gelation and emulsification, are used alongside more time-tested procedures to prepare nanoformulations. These methods are more difficult to implement and may call for more severe reaction conditions or the addition of other chemicals, both of which add to the expense of preparation. Electrostatic spinning and electrostatic spraying are two promising new technologies that could be used in the future to facilitate the more effective manufacture of nanoformulations through the application of straightforward procedures and moderate reaction conditions.

In conclusion, polyphenol-containing nanoformulations have a promising future in biomedical research, thanks to their malleable shapes, easy synthesis procedure, and low toxicity. If these issues can be resolved, then these nanoformulations will provide researchers with potent tools to tackle some of the most intractable scientific and technical difficulties in the biomedical profession. We hope this review will provide readers with a foundational understanding of the state of polyphenol-containing nanoformulations in biomedicine, encourage greater study in this area, and direct the creation of novel polyphenol-containing functional materials.

## 7. Conclusions

The phenolic compounds (EGCG, resveratrol, curcumin, quercetin) found in plants in high concentrations perform a wide range of beneficial biological functions. Furthermore, poor stability, poor solubility, and limited bioavailability significantly limit the utilization of these compounds in food and medicine. Nanoparticle encapsulation not only allows for more precise targeting and controllable release but also allows for the circumvention of these limits. Nanotechnology provides an ideal carrier system for increasing the pharmacokinetics and bioavailability of polyphenols. Nanoparticles are nearly ideal as carriers; however, their side effects and toxicity must be considered and mitigated before they may be used in a therapeutic setting. As polyphenols are natural compounds that must be taken for an extended period of time in the treatment and prevention of diseases, it is vital to understand the dangerous side effects linked to the buildup of nanoparticles in the physiological system. Specifically, if the nanoparticles have a poor encapsulation rate, this will be the case. Thus, standardized in vitro and in vivo models must be constructed, and in vivo safety testing procedures must be verified to support the development and implementation of innovative, effective nanoparticles beneficial to human health.

## Figures and Tables

**Table 1:** Overview of various polyphenols and their pharmacological effects.

Health Benefits	Polyphenol	Study Model	Effect	References
Anticancer	Epigallocatecin-3-gallate (EGCG)	Liver cancer	Inhibits the growth and induces apoptosis of different liver cancer cells	[2]
	Curcumin	Prostate cancer	Promotes apoptosis of the prostate cancer cells both through the mitochondrial and the receptor-mediated pathways	[18]
	Resveratrol	Bladder cancer	Inhibit cancer cell proliferation, cell migration, and invasion, induce cell cycle arrest, and trigger apoptosis	[19]
	Naringin	Pancreatic cancer	Inducing tumor cell death and inhibiting angiogenesis in malignant cells	[20]
Pro-oxidant	EGCG	Breast cancer	Inhibit tumorigenesis during the initiation, promotion and progression stages	[21]
	Curcumin	Prostate cancer	Quenches free radicals, induces antioxidant enzymes (catalase, superoxide dismutase, glutathione peroxidase)	[18]
	Resveratrol	*Saccharomyces cerevisiae*	Increase reactive oxygen species (ROS) generation through reverse electron transport	[22]
	Naringin	Prostate cancer	Suppress cell proliferation and trigger apoptosis in various cancer cell lines in the presence of copper ions	[20]
	Gallic acid	Breast cancer	Suppress cell proliferation and trigger apoptosis in various cancer cell lines in the presence of copper ions	[23]
	Genistein	Prostate cancer	Suppress cell proliferation and trigger apoptosis in various cancer cell lines in the presence of copper ions	[24]
Anti-inflammatory	Rutin	*Schistosomiasis mansoni*	Reduces pathological alterations	[25]
	Curcumin	Sunflower seed protein isolate	Blocks metabolic pathways leading to inflammation	[26]
	Resveratrol	Mice model	Inhibiting pro-inflammatory signaling pathways	[27]
Anti-hypertensive	Curcumin	Clinical trial	Prevents the transport of calcium, which aids in muscle cell contraction, causing artery dilatation	[28]
	Green tea catechins	Clinical trial	Improves endothelial function and insulin sensitivity, reduces blood pressure	[29]
	Genistein	Clinical trial	Significantly reduced the levels of total and low-density lipoprotein (LDL)-cholesterols and systolic blood pressure	[30]
Anti-diabetic	EGCG	In vitro	Modifies glucose and lipid metabolism in cells and markedly enhances glucose tolerance	[31]
	Curcumin	In vivo	Reduce blood sugar levels and increase insulin sensitivity	[32]
	Quercetin	Rats and mice	Lowers serum glucose in a dose dependent fashion	[33]
Anti-microbial	Curcumin	Viruses, bacteria, fungi	Blocks wide range of mechanistic pathways that are responsible for the microbial growth	[34]
	EGCG	*Staphylococcus aureus* and *Rhizoctonia solani*	Inhibits a wide variety of microbial growth-promoting metabolic processes	[35]

**Table 2:** Nanoencapsulation of nanostructures made of polyphenolic compounds, polysaccharides, and proteins (adapted from ref. [84]).

Polyphenol	Method	Encapsulating Material	Size (nm)	Efficiency	Outcome
Quercetin	Self-assembly	*Hohenbuehelia serotina* polysaccharides	360	Varies between 21–53%	Maintenance of stability and its anti-proliferative activities during in vitro GI digestion
Anthocyanin	Ionic gelation	Chitosan Beta Lactoglobulin	580	77%	Storage stability and oxidant stability during in vitro simulated digestion
Malvidin	Emulsification	Soybean insoluble dietary fiber	300	NA	Storage stability and protection in color
EGCG	Ionic gelation	Chitosan and Beta Lactoglobulin	100–500	60%	Release EGCG in GI tract
Anthocyanin	Self-assembly	Pectin—Whey Protein Isolate	200	55%	Improve stability
Olive leaf polyphenol	Nanoemulsion	Pectin—Whey Protein Concentrate	347	72–96%	Increased antioxidant properties and release rate
Pomegranate peel extract polyphenol	Nanoemulsion	Pectin—Cellulose	200	20%	Increased antimicrobial activity
Resveratrol	Antisolvent precipitation and electrostatic deposition	Pectin	120	NA	Stability, bioaccessibility, antioxidant capacity
Quercetin and Resveratrol	Antisolvent precipitation	Zein-Caboxymethyl cellulose	217	25%	Thermal stability
EGCG	Ionic cross linking	Carboxymethyl chitosan	400	75%	Increase antitumor activity

## Data Availability

Not applicable.
